# Neurological involvement in hospitalized children with SARS-CoV-2 infection: a multinational study

**DOI:** 10.1017/cjn.2022.347

**Published:** 2023-01-04

**Authors:** Carmen Yea, Michelle Barton, Ari Bitnun, Shaun K. Morris, Tala El Tal, Rolando Ulloa-Gutierrez, Helena Brenes-Chacon, Adriana Yock-Corrales, Gabriela Ivankovich-Escoto, Alejandra Soriano-Fallas, Marcela Hernandez-de Mezerville, Peter Gill, Alireza Nateghian, Behzad Haghighi Aski, Ali Anari Manafi, Rachel Dwilow, Jared Bullard, Jesse Papenburg, Rosie Scuccimarri, Marie-Astrid Lefebvre, Suzette Cooke, Tammie Dewan, Lea Restivo, Alison Lopez, Manish Sadarangani, Ashley Roberts, Jacqueline Wong, Nicole Le Saux, Jennifer Bowes, Rupeena Purewal, Janell Lautermilch, Cheryl Foo, Joanna Merckx, Joan Robinson, E. Ann Yeh

**Affiliations:** 1 Neuroscience and Mental Health, SickKids Research Institute, Toronto, Ontario, Canada; 2 Department of Pediatrics, University of Western Ontario, London, Ontario, Canada; 3 Department of Pediatrics, University of Toronto, Toronto, Ontario, Canada; 4 Division of Infectious Diseases, The Hospital for Sick Children, Toronto, Ontario, Canada; 5 Division of Rheumatology, The Hospital for Sick Children, Toronto, Ontario, Canada; 6 Department of Pediatrics, Hospital Nacional de Niños “Dr. Carlos Sáenz Herrera”, Caja Costarricense de Seguro Social (CCSS), San José, Costa Rica; 7 Division of Pediatric Medicine, The Hospital for Sick Children, Toronto, Ontario, Canada; 8 Department of Pediatrics, Iran University of Medical Sciences, Tehran, Iran; 9 Department of Pediatrics, University of Manitoba, Winnipeg, Manitoba, Canada; 10 Division of Pediatric Infectious Diseases, Dept. of Pediatrics, Montreal Children’s Hospital (McGill University Health Centre), Montreal, Quebec, Canada; 11 Division of Microbiology, Dept. of Clinical Laboratory Medicine, Optilab Montreal, McGill University Health Centre, Montreal, Quebec, Canada; 12 Division of Rheumatology, Montreal Children’s Hospital (McGill University Health Centre), Montreal, Quebec, Canada; 13 Department of Pediatrics, University of Calgary, Calgary, Alberta, Canada; 14 BC Children’s Hospital, Vancouver, BC, Canada; 15 Vaccine Evaluation Center, University of British Columbia, Vancouver, BC, Canada; 16 Department of Pediatrics, McMaster University, Hamilton, Ontario, Canada; 17 Department of Pediatrics, University of Ottawa, Ontario, Canada; 18 Children’s Hospital of Eastern Ontario Research Institute, Ottawa, Ontario, Canada; 19 Department of Pediatrics, University of Saskatchewan, Saskatoon, Saskatchewan, Canada; 20 Department of Pediatrics, Memorial University, St. John’s, Newfoundland and Labrador, Canada; 21 Department of Epidemiology, Biostatistics and Occupational Health, McGill University, Montreal, Quebec, Canada; 22 Department of Pediatrics, University of Alberta, Edmonton, Alberta, Canada; 23 Division of Neurology, The Hospital of Sick Children, Toronto, Ontario, Canada

**Keywords:** COVID-19, SARS-CoV-2, Neurologic, Seizures, Encephalopathy, Pediatrics, Children

## Abstract

**Background and Objectives:**

Neurological involvement associated with SARS-CoV-2 infection is increasingly recognized. However, the specific characteristics and prevalence in pediatric patients remain unclear. The objective of this study was to describe the neurological involvement in a multinational cohort of hospitalized pediatric patients with SARS-CoV-2.

**Methods:**

This was a multicenter observational study of children <18 years of age with confirmed SARS-CoV-2 infection or multisystemic inflammatory syndrome (MIS-C) and laboratory evidence of SARS-CoV-2 infection in children, admitted to 15 tertiary hospitals/healthcare centers in Canada, Costa Rica, and Iran February 2020–May 2021. Descriptive statistical analyses were performed and logistic regression was used to identify factors associated with neurological involvement.

**Results:**

One-hundred forty-seven (21%) of 697 hospitalized children with SARS-CoV-2 infection had neurological signs/symptoms. Headache (*n* = 103), encephalopathy (*n* = 28), and seizures (*n* = 30) were the most reported. Neurological signs/symptoms were significantly associated with ICU admission (OR: 1.71, 95% CI: 1.15–2.55; *p* = 0.008), satisfaction of MIS-C criteria (OR: 3.71, 95% CI: 2.46–5.59; *p* < 0.001), fever during hospitalization (OR: 2.15, 95% CI: 1.46–3.15; *p* < 0.001), and gastrointestinal involvement (OR: 2.31, 95% CI: 1.58–3.40; *p* < 0.001). Non-headache neurological manifestations were significantly associated with ICU admission (OR: 1.92, 95% CI: 1.08–3.42; *p* = 0.026), underlying neurological disorders (OR: 2.98, 95% CI: 1.49–5.97, *p* = 0.002), and a history of fever prior to hospital admission (OR: 2.76, 95% CI: 1.58–4.82; *p* < 0.001).

**Discussion:**

In this study, approximately 21% of hospitalized children with SARS-CoV-2 infection had neurological signs/symptoms. Future studies should focus on pathogenesis and long-term outcomes in these children.

## RÉSUMÉ

Atteinte neurologique chez des enfants hospitalisés pour une infection à SRAS-CoV-2 : résultats d’une étude multinationale.

**Contexte et objectif:**

L’atteinte neurologique associée à l’infection à SRAS-CoV-2 (coronavirus 2 du syndrome respiratoire aigu sévère) est de plus en plus reconnue. Toutefois, on n’en connaît pas très bien la prévalence ni les caractéristiques particulières chez les enfants. L’étude visait donc à faire état de l’atteinte neurologique associée à l’infection à SRAS-CoV-2 dans une cohorte multinationale d’enfants hospitalisés.

**Méthode:**

Il s’agit d’une étude d’observation multicentrique, réalisée chez des enfants < 18 ans souffrant d’une infection à SRAS-CoV-2 avérée ou du syndrome inflammatoire multisystémique (SIME [E : enfants]) associé à des signes d’infection à SRAS-CoV-2 confirmés en laboratoire; la cohorte se composait d’enfants traités dans 15 centres hospitaliers de soins tertiaires ou centres de santé, au Canada, au Costa Rica ou en Iran, de février 2020 à mai 2021. Ont été réalisées des analyses statistiques descriptives ainsi qu’une analyse de régression logistique permettant de dégager des facteurs associés à l’atteinte neurologique.

**Résultats:**

Sur 697 enfants hospitalisés pour une infection à SRAS-CoV-2, 147 (21 %) présentaient des signes ou des symptômes d’atteinte neurologique. Les céphalées (*n* = 103), les encéphalopathies (*n* = 28) et les crises d’épilepsie (*n* = 30) étaient les manifestations les plus fréquentes. Les signes et symptômes d’atteinte neurologique étaient significativement associés aux admissions aux unités de soins intensifs (USI) (RRA [risque relatif approché] : 1,71; IC à 95 % : 1,15–2,55; *p* = 0,008), au respect des critères du SIME (RRA : 3,71; IC à 95 % : 2,46–5,59; *p* < 0,001), à la fièvre durant le séjour à l’hôpital (RRA : 2,15; IC à 95 % : 1,46–3,15; *p* < 0,001) et aux troubles gastro-intestinaux (RRA : 2,31; IC à 95 % : 1,58–3,40; *p* < 0,001). Par ailleurs, les manifestations d’atteinte neurologique différentes des céphalées étaient significativement associées aux USI (RRA : 1,92; IC à 95 % : 1,08–3,42; *p* = 0,026), aux troubles neurologiques sous-jacents (RRA : 2,98; IC à 95 % : 1,49–5,97; *p* = 0,002) et aux antécédents de fièvre avant l’hospitalisation (RRA : 2,76; IC à 95 % : 1,58–4,82; *p* < 0,001).

**Discussion:**

D’après les résultats de l’étude, environ 21 % des enfants hospitalisés pour une infection à SRAS-CoV-2 présentaient des signes ou des symptômes d’atteinte neurologique. Il faudrait que la pathogenèse des troubles neurologiques et les résultats cliniques à long terme chez les enfants touchés fassent l’objet d’études futures de recherche.

## Introduction

Although the site of primary infection in severe acute respiratory syndrome coronavirus-2 (SARS-CoV-2) is the respiratory system, there is accumulating laboratory and clinical evidence that suggests that SARS-CoV-2 may have neurotropic and/or neuroinvasive properties^1–4^ similar to other human coronaviruses.^5–9^ Neurological involvement associated with SARS-CoV-2 infection have been described in adults, ranging from non-specific neurologic symptoms such as myalgia, dizziness, and headache,^10,11^ to more severe neurological complications including acute encephalopathy, cerebrovascular disorder, stroke, Guillain-Barré Syndrome, and peripheral nervous system disorders. They occur in up to 82% of adult COVID-19 patients.^12–16^

In children, SARS-CoV-2 infection generally follows a milder disease course acutely as compared to adults, but several case series have documented severe signs/symptoms in some children^17,18^ Furthermore, case series in Europe and the United States (US) have highlighted the presence of neurological complications in some children.^19–22^ Risk factors for neurological involvement in hospitalized children are not firmly established. To address this gap, we evaluated (1) neurological signs/symptoms temporally associated with SARS-CoV-2 infection in hospitalized children in a multicenter, multinational cohort, and (2) associations between neurological involvement, pre-existing comorbidities, and clinical outcomes.

## Methods

### Study Design and Population

This was a multinational, retrospective study of a prospectively collected cohort of consecutive patients (0–18 years of age) admitted for any medical reasons from February 1, 2020 through May 31, 2021 who had laboratory-confirmed SARS-CoV-2 infection. The study cohort comprised of hospitalized children from 15 centers: 13 tertiary hospitals that were part of the Pediatric Investigators Collaborative Network on Infections in Canada (PICNIC), and one hospital each in San José, Costa Rica, and Tehran, Iran. Study size was determined by the number of children admitted meeting study criteria during the period of the study. SARS-CoV-2 infection was confirmed by positive reverse transcription-polymerase chain reaction (RT-PCR) of any clinical specimen (nasopharyngeal swab, throat swab, nose swab, endotracheal tube aspirates) and/or positive serological testing for antibodies against SARS-CoV-2. Routine clinical evaluations and laboratory measurements were obtained as part of standard of care and were performed according to institutional protocols. Patients were managed at the discretion of the treating physicians locally.

### Standard Protocol Approvals, Registrations, and Patient Consents

Ethics approval was obtained according to local ethics committee requirements.

### Clinical Data Collection

Chart review and data entry were performed locally at each participating center by designated research staff. Study data were abstracted from clinical charts using standardized case report forms (available upon request) and definitions, and de-identified data were entered into an electronic data capture tool (REDCap) hosted at the University of Alberta.^23^ Study data regarding demographics, medical history, comorbidities, clinical features, laboratory evaluations, neuroimaging (brain and spine magnetic resonance imaging (MRI)), and treatment regimen were collected.

### Clinical Definitions

Children hospitalized for any medical reasons and had lab-confirmed SARS-CoV-2 infection (with or without neurological signs/symptoms) were included in this study. We defined a neurological sign/symptom as the presence of any of the following: (1) new or worsening seizures, encephalopathy (altered/decreased level of consciousness, personality/behavioral change), psychosis, ataxia, dyskinesia, muscle weakness, sensory abnormalities, bulbar symptoms (facial weakness, dysarthria, dysphagia), headache; (2) abnormal CSF WBC (>5 WBC/mm^3^) or protein (>0.4 g/L) or (3) new/acute central nervous system MRI abnormalities. We defined encephalopathy in children 1 year of age and older as having altered level of consciousness with or without irritability/lethargy, whereas in neonates/infants younger than 1 year of age we also included irritability and/or lethargy regardless of change in consciousness given the difficulty in establishing level of consciousness in this age group. Categorization of MIS-C was made using clinical data provided by site Principal Investigators. For the purpose of data analyses in this study, children were retrospectively classified as MIS-C if they met the World Health Organization (WHO) criteria for MIS-C in relation to systemic symptoms^24^ with an additional modification to include those with fever for less than 2 days if corticosteroids or intravenous immunoglobulin (IVIG) was administered prior to day 3 of fever. Children with ADEM in absence of a purely systemic syndrome prior who were given steroids were not classified as MIS-C. Cases of neurological manifestations who were hospitalized for primary reasons other than COVID-19 and had incidental SARS-CoV-2 infection were adjudicated by a blinded pediatric neurologist (EAY). Cases of neurological manifestations that had potential pathogens detected in addition to SARS-CoV-2 were adjudicated individually by site investigators and secondarily by a blinded infectious disease specialist (AB): neurological involvement in these cases was categorized as 1) most likely due to SARS-CoV-2 infection (COVID-19/MIS-C), 2) most likely due to another pathogen, or 3) indeterminate in which the role of SARS-CoV-2 is uncertain. To avoid ambiguity in evaluating the association of SARS-CoV-2 and neurological signs/symptoms, cases of neurological manifestation that were deemed to be most likely due to other pathogens or indeterminate (categories 2 and 3 above) were excluded. Neuroimaging was performed at the discretion of the attending physician. MRI data included descriptive information gleaned from clinical reports. MRI reports were reviewed by a pediatric neurologist with special expertise in neuroinflammation (EAY), and findings were classified as 1) normal; 2) acute in the context of SARS-CoV-2 (bright signal on T2 weighted imaging ± enhancement, ± diffusion restriction in the absence of alternative chronic explanations for the abnormalities); 3) non-acute, which included those that showed chronic, incidental findings (e.g. cortical dysplasia, neoplasm).

### Statistical Analysis

Continuous data were presented as median (interquartile range [IQR]) and compared using non-parametric Mann–Whitney *U* test. Categorical data were described using counts (percentage) and compared using chi-square or Fisher’s exact test, as appropriate. Correlation analysis was performed using Spearman’s rho test. A *p*-value less than 0.05 was considered statistically significant. Logistic regression was used for analysis of factors associated with neurological involvement. Imputations were not done for any missing data. Due to sample size limitations, only descriptive analysis was performed in two subgroups: 1) children with MIS-C and 2) children with neurological signs/symptoms and no overt infectious symptoms. Benjamini-Hochberg false discovery rate <0.05 was used to adjust for multiple comparisons. JASP (version 0.14) was used for statistical analysis.

### Data Availability

The data that support findings in this study are available upon reasonable request from the Corresponding Author. Data are not publicly available as they contain information that could comprise the privacy of patients.

### Role of Funding Source

There was no funding source for this study. The Corresponding Author had full access to all the data in the study and had final responsibility for the decision to submit for publication.

## Results

### Study Cohort

This multicenter study included 713 hospitalized children (413 [58%] male, median 4 years of age [IQR 0.8–10.5]) with laboratory evidence of SARS-CoV-2 infection (Canada, *n* = 13; Costa Rica, *n* = 1; Iran, *n* = 1; Table 1). In 28 of these children, one or more other possible pathogens were detected. Sixteen of these 28 were excluded from further analysis: 13 where another pathogen was thought to be the more likely cause of neurologic signs/symptoms, and 3 because there was causal uncertainty (Supplementary Figure). In the remaining 12 cases, SARS-CoV-2 was deemed the more likely cause of neurologic signs/symptoms. Thus, the final cohort consisted of 697 children (Table 1). Patients from Canada, Costa Rica, and Iran constituted 62, 29, and 8% of the study cohort, respectively (Table 1). Patient demographics and clinical features stratified by country are summarized in Supplementary Table 1. The Canadian cohort was significantly older (6 years [IQR 1.32–13.8]) than those from Costa Rica (2.7 years [IQR 0.5–7.5] and Iran (2.1 years [IQR 0.7–5.3]; *p* < 0.001).

**Table 1:** Demographics, clinical characteristics, treatment, and outcomes of children with and without neurological involvement

	All patients (*n* = 697)	Any neurological involvement (*n* = 147)	No neurological involvement (*n* = 550)	*p*-value
**Demographics**				
Age at admission, years	4 (0.9-10.5)	7.3 (3-12.2)	3.3 (0.7-10.3)	** <0.001**
Male	403 (58%)	89 (61%)	314 (57%)	0.451
Female	294 (42%)	58 (39%)	236 (43%)	0.451
Country				
Canada	435 (62%)	91 (62%)	344 (62%)	0.887
Costa Rica	205 (29%)	44 (30%)	161 (29%)	0.876
Iran	57 (8%)	12 (8%)	45 (8%)	0.994
**Underlying conditions**				
Previously healthy	390 (56%)	89 (60%)	301 (55%)	0.207
Preterm birth (<37 weeks GA)	45 (6%)	3 (2%)	42 (8%)	**0.014**
Congenital immunodeficiency	4 (0.6%)	0	4 (1%)	0.300
Cancer	36 (5%)	5 (3%)	31 (6%)	0.277
Cardiovascular disease	19 (3%)	0	19 (3%)	0.300
Asthma	46 (7%)	11 (7%)	35 (6%)	0.627
Cystic fibrosis	2 (0.3%)	0	2 (0.3%)	0.464
Chromosomal disorder	11 (2%)	2 (1%)	9 (2%)	0.166
Diabetes	11 (2%)	1 (0.7%)	10 (2%)	0.812
Hypertension	9 (1%)	3 (2%)	6 (1%)	0.365
Obesity	57 (8%)	12 (8%)	45 (8%)	0.994
Neurological disorder	74 (10%)	19 (13%)	55 (10%)	0.306
Seizure disorder	40 (6%)	12 (8%)	28 (5%)	0.155
Developmental delay/autism	38 (5%)	7 (5%)	31 (6%)	0.678
Cerebral palsy	8 (1%)	2 (1%)	6 (1%)	0.785
Other complex/chronic neurological disorders	14 (2%)	5 (3%)	9 (2%)	0.846
≥2 comorbid conditions	92 (13%)	20 (14%)	72 (13%)	0.733
**Primary reason for hospital admission**				
COVID-19/probable MIS-C	424 (61%)	108 (73%)	316 (57%)	**0.002**
Other reasons/COVID-19 did not prolong hospital stay	188 (27%)	14 (9%)	174 (32%)	** <0.001**
Other reasons but COVID-19 prolonged hospital stay	45 (6%)	11 (7%)	34 (6%)	0.960
MIS-C with fewer than 5 days of fever	11 (1%)	0	11 (2%)	0.345
**Presenting symptoms**				
Fever at any time during illness^a^	466 (67%)	124 (84%)	342 (62%)	** <0.001**
Fever prior to admission only	156 (22%)	38 (26%)	118 (21%)	0.223
Fever during admission	310 (44%)	86 (59%)	224 (41%)	** <0.001**
Duration of fever, days	4 (1-7)	6 (4-8)	4 (1–7)	** <0.001**
Rash	127 (18%)	45 (31%)	82 (15%)	** <0.001**
Respiratory symptoms	408 (58%)	88 (60%)	320 (58%)	0.713
Gastrointestinal symptoms	329 (47%)	95 (64%)	234 (43%)	** <0.001**
Myalgias	90 (13%)	52 (35%)	38 (7%)	** <0.001**
Conjunctivitis	117 (17%)	49 (33%)	68 (12%)	** <0.001**
Anosmia or ageusia	22 (3%)	9 (6%)	13 (2%)	**0.006**
Syncope	7 (1%)	4 (3%)	3 (0.5%)	**0.007**
Edema of hands and feet	56 (8%)	25 (17%)	31 (6%)	** <0.001**
Redness of hands and feet	50 (7%)	21 (14%)	29 (5%)	** <0.001**
Peeling of fingers and toes	6 (1%)	1 (0.6%)	5 (0.9%)	0.790
Cracked red lips	69 (10%)	34 (23%)	35 (6%)	** <0.001**
Strawberry tongue	49 (7%)	29 (20%)	20 (4%)	** <0.001**
Enlarged lymph node	31 (4%)	13 (9%)	18 (3%)	**0.002**
Fatigue	23 (3%)	10 (7%)	13 (2%)	**0.017**
Met MIS-C criteria	140 (20%)	60 (41%)	80 (14%)	** <0.001**
**Treatment**				
Antivirals^b^	58 (8%)	18 (12%)	40 (7%)	0.053
Antibiotics^c^	122 (17%)	25 (17%)	97 (18%)	0.351
Hydroxychloroquine	35 (5%)	9 (6%)	26 (5%)	0.491
IL-6 inhibitor (Tocilizumab)	11 (1%)	2 (1%)	9 (2%)	0.812
IL-1 receptor antagonist (Anakinra)	6 (0.8%)	3 (2%)	3 (0.5%)	0.081
IVIG and steroids	100 (14%)	44 (30%)	56 (10%)	** <0.001**
IVIG only	49 (7%)	18 (12%)	31 (6%)	**0.005**
Steroids only	148 (21%)	28 (19%)	120 (22%)	0.446
None of the above	365 (52%)	51 (35%)	313 (57%)	** <0.001**
ICU	176 (25%)	51 (35%)	125 (23%)	**0.003**
Mechanical ventilation	114 (16%)	31 (21%)	83 (15%)	0.104
Duration of hospital stay, days	4 (2–8)	5 (3–9)	4 (2–7)	**0.005**

Abbreviations: ICU, intensive care unit; IL, interleukin; IVIG, intravenous immunoglobulins; MIS-C, multisystem inflammatory syndrome in children.

a Data missing for 12/697 patients.

b Oseltamivir, Kaletra, Remdesivir.

c Antibiotics for possible secondary bacterial pneumonia.

SARS-CoV-2 infection was confirmed by RT-PCR in 581/697 (83%) children and by serology assay in 77/693 (11%). Thirty-nine (5%) children tested positive by both RT-PCR and serology. One-hundred and eighty-eight (27%) children were hospitalized with primary reasons other than SARS-CoV-2 infection but had tested positive for SARS-CoV-2 on routine screening; of these, 44 did not have symptoms typical of SARS-CoV-2 infection.

### Neurological Signs/Symptoms in Overall Cohort

Table 1 shows the clinical features of children with and without neurological involvement. Of the 697 hospitalized children, 147 (21%) had at least one neurological sign/symptom temporally associated with SARS-CoV-2 infection and 40 (6%) presented with 2 or more neurological signs/symptoms. The most common neurological signs/symptoms were headache (15% [103/697]), acute encephalopathy (4% [28/697]), and seizures (4% [30/697]; Table 2).

**Table 2:** Prevalence of neurological manifestations in 697 hospitalized children with confirmed SARS-CoV-2 infection

Neurological symptoms	*n* (%)
Headache^a^	103 (15%)
Encephalopathy^b^	28 (4%)
Seizures	30 (4%)
Altered mental status	14 (2%)
Lethargy^c^	16 (2%)
Irritability^c^	8 (1%)
Sensory abnormality	3 (0.4%)
Speech impairment	3 (0.4%)
Truncal weakness	1 (0.1%)
Facial weakness	1 (0.1%)

a 85 patients had headache solely as their neurological symptoms; 12 patients had headache in addition to other neurological symptoms.

b Includes 18 infants <1 year old presenting with irritability and/or lethargy regardless of LOC change.

c Includes only patients >1 year of age.

Twelve percent (85/697) of children had headache only and no other neurological involvement. We performed a subgroup analysis comparing children with headache only (*n* = 85) versus those with other neurological signs/symptoms (*n* = 62) and found that children with headache only were significantly older (9.1 years [IQR 6-14] vs 2.5 years [0.3–9.6]; *p* < .0001) and had increased frequency of gastrointestinal symptoms (83% vs 39%; *p* < 0.001) and fever during admission (74% vs 37%; *p* < 0.001). We also noted that more children who had headache only met MIS-C criteria as compared to those who had non-headache neurological involvement (62% vs 11%; *p* < 0.001). The frequency of ICU admission (33% vs 37%; *p* = 0.601), length of ICU (5 [IQR 4–13] vs 3.5 [IQR 2–6]; *p* = 0.316) and length hospital stay (6 [IQR 4–8] vs 5 [IQR 3–12.5]; *p* = 0.786]) for children with headache only and those who had other neurological signs/symptoms were not significantly different (Supplementary Table 2).

Acute encephalopathy/altered mental status was reported in 28/697 (4%) of children. Of these, 15/28 (53%) were infants less than one year of age, and 5/28 (18%) children met MIS-C criteria. Ten of 28 (36%) children were admitted to the ICU and 6 (including 2 infants) needed mechanical ventilation. Four of 5 children with MIS-C and acute encephalopathy/altered mental status were admitted to the ICU with one requiring mechanical ventilation.

**Figure 1:**
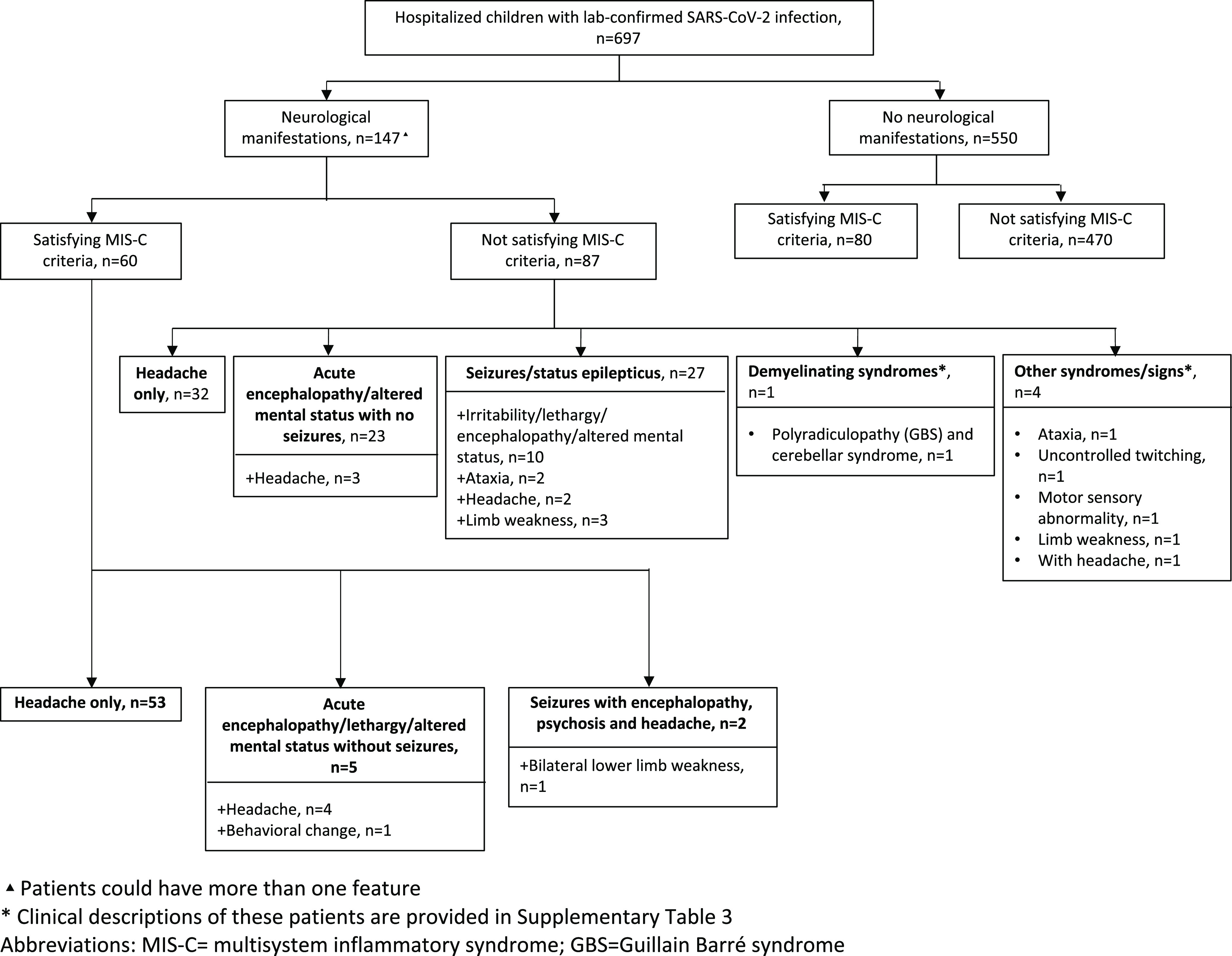
Neurological classification of study cohort.

Seizures, including status epilepticus, were reported in 29/697 (4%) of our cohort. Twelve of 29 (41%) patients with seizures had an underlying seizure disorder and two met MIS-C criteria. Thirteen (45%) of 29 patients with seizures were admitted to the ICU and 4 (14%) needed mechanical respiratory support. Of the 17 patients with seizures but who had no history of seizure disorder, 9 (53%) had at least one other neurological sign/symptom, including acute encephalopathy/altered mental status (*n* = 5), headache (*n* = 4), limb weakness (*n* = 3), and ataxia (*n* = 2; Figure 1).

In a subgroup analysis in which we excluded children who had headache as the only symptom, we found higher frequency of neurological involvement among children with an underlying seizure disorder (30% [12/40]) than those who had no history of a seizure disorder (9% [50/572]; *p* < 0.001). Notably, all 12 children with an underlying seizure disorder and who also had neurological involvement presented with seizures.

Five children, not included in the above categories, had other neurological involvement including polyradiculopathy (Guillain-Barré Syndrome) and cerebellar syndrome (*n* = 1), weakness (*n* = 1), abnormal movements including twitching (*n* = 1), motor sensory abnormality (*n* = 1), and ataxia (*n* = 1).

### Neurological Signs/Symptoms in Children with MIS-C

Overall, 20% (140/697) of patients satisfied MIS-C criteria. Children with MIS-C were significantly older (median 7.2 years [IQR 4–10] vs 2.7 [IQR 0.4–11]; *p* < 0.001) and fewer had an underlying neurological condition (2% [5/140] vs 12% [69/557]; *p* = 0.002) compared to children who did not meet MIS-C criteria. Children with neurological involvement in the MIS-C cohort were less likely than those in the non-MIS-C cohort to have a history of an underlying medical condition (MIS-C 22% vs non-MIS-C 52%; *p* < 0.001).

Sixty of 140 children who satisfied MIS-C criteria had neurological signs/symptoms, with headache (42% [59/140]), acute encephalopathy/altered mental status/psychosis (5% [7/140]), and seizures (1% [2/140]) being the most common. Fifty-three (38%) of 140 children with MIS-C experienced headache only while seven (5%) children who met MIS-C had at least one neurological sign/symptom other than headache (Figure 1). Sixty-six (47%) children with MIS-C were admitted to the ICU, of whom 30 had neurological involvement.

When compared to children who had neurological involvement and did not satisfy criteria for MIS-C, those with MIS-C were more likely to have headache alone (MIS-C 88% [53/60] vs non-MIS-C 37% [32/87]). When headache is removed from the analysis, the association is in the opposite direction: 7/140 (5%) patients satisfying MIS-C criteria developed neurological signs/symptoms vs. 55/557 (10%) non-MIS-C.

### Asymptomatic SARS-CoV-2 and Neurological Events

Of the entire study cohort, 5/697 (0.7%) had neurological signs/symptoms only with no other typical COVID-19 symptoms.

### Comparisons of Patients With and Without Neurological Involvement

Table 1 summarizes the clinical characteristics of children with and without neurological involvement. When compared to children without neurological involvement, those who had any neurological signs/symptoms were significantly older (7.3 years [IQR 3–12] vs 3.3 [IQR 0.7–10.30]; *p*<0.001), were more likely to be febrile at the time of admission or while hospitalized (60% [86/143] vs 41% [224/542]; *p* < 0.001), remained febrile for longer periods (median 6 days [IQR 4–8] vs 4 days [IQR 1–7]; *p* < 0.001), were more likely to meet MIS-C criteria [41% [60/147] vs 14% [80/550]; *p* < 0.001), more frequently reported symptoms associated with MIS-C, were more likely to be admitted to the ICU (35% [51/147] vs 23% [125/550]; *p* = 0.003), and had longer hospital stays (median 5 days [IQR 3–9] vs 4 days [IQR 2-7]; *p* < 0.005; Table 1). However, it is important to note that when patients with headache as their sole neurological sign/symptom were not included in the neurological group, except for the frequency of ICU admission which remained significantly higher (37% [23/62] vs 23% [125/550]; *p* = 0.012), the above-described differences were no longer significant.

### CSF Findings

Twenty-seven (17%) of 163 children with any neurological involvement had a lumbar puncture performed within a median of 2.5 days (IQR 2–7) from COVID-19 symptom onset (Table 3). None of the 27 CSF samples examined had detectable SARS-CoV-2 by RT-PCR. CSF analysis showed pleocytosis (>5 WBC/mm^3^) in 8/27 patients (lymphocyte predominance in 7/27 and neutrophil predominance in 1/27) and elevated protein in 10/27 (median 29 mg/dL [IQR 21-88 mg/dL].

**Table 3:** CSF evaluation of samples from patients with neurological manifestations^a^

CSF pleocytosis (>5 cells/mm^3^)	5 (23%)
Elevated CSF protein	4 (18%)
Median days from symptom onset to lumbar puncture (IQR)	4 (2–7)

Abbreviations: CSF, cerebrospinal fluid; IQR, interquartile range.

a Data was available from 27 patients with non-headache neurological signs/symptoms.

### Neuroimaging

Sixteen of 73 patients with non-headache neurological involvement received MRI scans of the brain and 5 also received MRI scans of the spine. MRI abnormalities were found in 81.2% (12/16) of these children (12/16 MRI brain, 3/5 spine), of which 68.7% (10/16) had acute abnormalities. The remaining two had chronic incidental findings including a pituitary adenoma and focal cortical dysplasia.

Six of 10 MRI abnormalities in the brain that were acute in the context of SARS-CoV-2 infection demonstrated findings consistent with neuroinflammation, including patchy multifocal lesions distributed throughout the brain, brainstem, and deep gray matter, at times associated with diffusion restriction, leptomeningeal enhancement, or punctate areas of micro hemorrhage. The four other brain MRI scans with acute abnormalities included (*n* = 1) findings suggestive of posterior reversible encephalopathy syndrome and (*n* = 2) focal abnormalities including diffusion restriction in the caudate head, and possible cortical vein thrombosis (increased susceptibility in the area of a cortical vessel). All three spinal cord abnormalities were deemed acute. They included (a) (*n* = 1) longitudinally extensive lesion (C2-6) with associated swelling (*n* = 1) and (b) enhancement of the cauda equina and nerve roots (brain MRI was noted to show cerebellar lesions as well) (*n* = 1). Supplementary Table 3 provides information on the clinical features seen in these patients.

### Treatment

Sixty-one percent (90/147) of children with any neurological involvement received immunotherapy compared to 38% (207/550; *p* < 0.001) of those without. Sixteen (11%) of 147 of patients with neurological involvement received immunotherapy plus antivirals (oseltamivir, lopinavir/ritonavir, remdesivir). Of the 90 patients with neurological symptoms who received immunotherapy, 18(20%) received IVIG only, 28 (31%) received corticosteroids only and 44 (49%) received IVIG in combination with corticosteroids. Additionally, 2/147 children received IL-6 blocker (Tocilizumab) and 3/147 received IL-1 receptor antagonist (Anakinra; Table 1).

Fifty-one (85%) of 60 MIS-C patients with neurological signs/symptoms received immunotherapy. Of the patients who did not satisfy MIS-C criteria, 13/32 (41%) patients with headache only received immunotherapy; 7/23 (30%) patients with acute encephalopathy with no seizures received immunotherapy; 10/27 (37%) patients with seizures received immunotherapy; 2/5 (40%) patients with demyelinating and other syndromes received immunotherapy.

### Short-term Outcomes in Children with Non-headache Neurological Involvement

Data regarding neurological evaluation at hospital discharge were available for 37/62 (60%) of children with non-headache neurological signs/symptoms. Of these, 78% (29/37) reported no significant neurological deficits or had complete recovery while 22% (8/37) still had neurological deficits, with 5 children reporting residual motor deficits/limb weakness, one child continued to have increased seizures activity, and one had hypotonia (one case did not specify residual symptoms).

### Factors Associated with Neurological Signs/Symptoms

We performed multivariable logistic regression analyses to identify potential factors associated with any neurological involvement. Adjusting only for age and using separate models, we found that ICU admission (OR: 1.71, 95% CI: 1.15–2.55; *p* = 0.008), satisfaction of MIS-C criteria (OR: 3.71, 95% CI: 2.46–5.59; *p* < 0.001), fever during hospitalization (OR: 2.15, 95% CI: 1.46–3.15; *p* < 0.001), and gastrointestinal involvement (OR: 2.31, 95% CI: 1.58–3.40; *p* < 0.001) were significantly associated with any neurological involvement. We further performed a subgroup logistic regression analysis that included only patients who had neurological signs/symptoms other than headache given the high prevalence of headache among older patients and among MIS-C cases which could potentially introduce bias. After adjusting for age, we found that non-headache neurological signs/symptoms were significantly associated with ICU admission (OR: 1.92, 95% CI: 1.08–3.42; *p* = 0.026). We also found that underlying neurological disorders (OR: 2.98, 95% CI: 1.49–5.97, *p* = 0.002) and history of fever prior to hospital admission (OR: 2.76, 95% CI: 1.58–4.82; *p* < 0.001) were significantly associated with neurological involvement other than headache.

## Discussion

Approximately 21% of children hospitalized for SARS-CoV-2 infection in our study had neurological involvement. Among these, headache (15%), encephalopathy (4%), and seizures (4%) were most commonly reported, consistent with previous studies.^20,25^ Notably, headache constituted 70% of the patients with neurological involvement in our cohort. Subgroup analyses revealed that these patients who had headache solely as their neurological signs/symptoms were older and were more likely to meet MIS-C criteria (61% vs 11%) than children without headache, which corroborates with a recent study of 1493 children in the US who were diagnosed with SARS-CoV-2 or MIS-C.^25^ where approximately 47% of children diagnosed with MIS-C and 16% children with acute SARS-CoV-2 presented with headache. The reasons for this are likely complex, and we emphasize that headache may be a symptom only with no associated abnormalities on neurological examination or associated structural abnormalities in the brain as seen on MRI. The fact that the children with MIS-C were less likely to have neurological involvement than the non-MIS-C children may point to the lower rate of underlying neurological conditions in non-MIS-C cohort (12%) vs. the MIS-C cohort (2%) and presence of underlying medical diagnoses in the children in the non-MIS-C cohort (52%) vs. the MIS-C cohort (22%; *p* < 0.001).

We also make specific note of a high rate (12%) of neurological comorbidities in children with non-headache neurological signs/symptoms. In addition, we found that children with any neurological involvement were more likely to have ICU admission (OR 1.71), and children with non-headache neurological signs/symptoms were more likely to have underlying neurological disorders (OR 2.98) as were those with fever prior to admission (OR 2.76). This emphasizes the complex nature of the illness trajectory of children manifesting with neurological complications.

Seizures were common in our cohort – 20% of those with neurologic involvement and about 4% of the whole cohort – similar to the numbers that some have described in children hospitalized with SARS-CoV-2 infection (0.3-8%)^19,20,25–28^ and at least twice the rate of seizures in hospitalized adults (0-2%)^11,29,30^ Much of this can be explained by the relatively high proportion of children with underlying neurological disorders in our cohort with neurological complications and the relatively high rate of children with underlying seizure disorders. It is possible that many children suffered from neurological complications as a result of their overall medical complexity, signaled by the presence of an underlying neurological diagnosis. As this study did not capture specifics regarding medical complexity, we are unable to comment on how many of these children fell into the category of what could be defined as medically complex.

Of note, ICU admission rates appear relatively low among patients with encephalopathy in our cohort (36%), but when only children greater than 12 months of age were included in this calculation, 61% with encephalopathy required ICU admission. We emphasize that the decision to admit to ICU is center-based and that many centers would choose to admit patients to ICU only if the child was in need of intubation or higher-level respiratory support. Furthermore, children under 12 months of age were classified as encephalopathic if they exhibited lethargy and/or irritability. This likely led to the differences noted in rates of ICU stay between infants and children with encephalopathy.

The mechanisms by which neurological complications occurred in our cohort are unknown but are likely multifactorial. It is less likely that SARS-CoV-2 played a direct role in their neurological involvement, but their susceptibility to coinfection may have been enhanced by the presence of illness due to infection with SARS-CoV-2.^31^ As well, some of the seizures may have occurred as a non-specific response to fever or infection, as is seen frequently in the pediatric population. Finally, seven of the children had inflammatory CNS problems, similar to that which has been described elsewhere in the literature, and which are likely due to secondary immune mechanisms. We emphasized that these were not more frequently found in children with MIS-C suggesting that CNS abnormalities can occur in the absence of evidence of marked systemic inflammation.

Those manifestations, based on laboratory or MRI evidence, which were considered to be post- or para-infectious inflammatory neurological complications, were relatively infrequently seen, comprising just over 10% of the cohort with non-headache neurological complications. SARS-CoV-2 is known to gain entrance into host cells via the binding of viral spike protein to the angiotensin-converting enzyme-2 receptor that is expressed in almost all human tissues and is highly abundant in the brain.^2,4,32,33^ However, as in our study, the presence of viral RNA and protein has only been rarely identified in the brain and cerebrospinal fluid (CSF) of patients with COVID-19.^3,34,35^ While reports that SARS-CoV-2 can productively infect and replicate in human neuronal cells^36^ and in brain organoids raises the possibility of direct viral involvement of SARS-CoV-2 in the CNS,^4,32^ we emphasize that SARS-CoV-2 was not isolated in any CSF samples. At least some of these events therefore may have resulted from the immunological response to SARS-CoV-2 rather than direct viral invasion of the CNS.

Finally, in contrast to adult studies which document relatively high rates of CNS thrombotic events, only one child had a CNS thrombotic complication, and this was related to a superficial cortical vein. No children presented with stroke or sinovenous thrombosis. This is in keeping with other studies of children which report relatively low rates of CNS thrombotic complications compared to adult populations.^11,19,20,37–39^

Importantly, abnormalities were likely to be found on neuroimaging in the small number of children who received brain and spine imaging: over 75% of the MRI scans of the brain/spine which were performed were abnormal. Most abnormalities (9/16) appeared to be acute in the context SARS-CoV-2 as described in the results, but interestingly, SARS-CoV-2 infection may have unmasked unrelated neurologic diagnoses, such as presentation with a cluster of new-onset seizures in a child with underlying cortical dysplasia.

Our study’s strengths include the comprehensive coverage of Canadian institutions, multinational collaboration, the relatively large cohort, and the presence of a detailed neurologically focused case report form. We acknowledge several limitations, however. First, the study was retrospective, posing the risk of selection bias, although it is likely that most hospitalized children with positive SARS-CoV-2 testing were captured in the participating centers due to strong surveillance measures, reporting, and frequent testing in each of the participating centers. Second, in-hospital management strategies may differ between institutions, affecting clinical outcome variables including frequency of MRI and CSF acquisition, length of hospital stay, and ICU admission. Third, the aggregate nature of data from various institutions across different countries (all of which participated in this study voluntarily) as well as small sample size restricts the generalizability of findings. Fourth, due to the nature of the data collection, we are unable draw conclusions about the etiologies of the neurological involvement, including potential neurological signs/symptoms which do not have a specific etiological tie to SARS-CoV-2, such as febrile convulsions. Fifth, the classification of encephalopathy in children younger than 1 year of age is challenging, and decision-making around classification is subjective. Sixth, as the majority of infants under one who were noted to have neurological involvement were classified as having encephalopathy, it is possible that some of these children had non-specific symptoms related to general illness rather than focal, neurological abnormalities. Lastly, it is possible that for some of the patients with coinfection, the CNS manifestations and/or abnormal imaging findings were entirely due to the coinfection.

## Conclusion

We observed a relatively high rate of neurological involvement in children hospitalized with SARS-CoV-2 infection. Further studies are needed to confirm associations between these factors and neurological involvement of SARS-CoV-2 and investigate long-term neurological and cognitive outcomes in these children.

## Supplementary material

For supplementary material accompanying this paper visit https://doi.org/10.1017/cjn.2022.347. click here to view supplementary material

## References

[1] Baig AM , Khaleeq A , Ali U , Syeda H. Evidence of the COVID-19 virus targeting the CNS: tissue distribution, host-virus interaction, and proposed neurotropic mechanisms. ACS Chem Neurosci. 2020;11:995–8. 32167747 10.1021/acschemneuro.0c00122

[2] Lu R , Zhao X , Li J , et al. Genomic characterisation and epidemiology of 2019 novel coronavirus: implications for virus origins and receptor binding. Lancet. 2020;395:565–74. 32007145 10.1016/S0140-6736(20)30251-8 PMC7159086

[3] Moriguchi T , Harii N , Goto J , et al. A first case of meningitis/encephalitis associated with SARS-Coronavirus-2. Int J Infect Dis IJID Off Publ Int Soc Infect Dis. 2020;94:55–8. 10.1016/j.ijid.2020.03.062 PMC7195378 32251791

[4] Pellegrini L , Albecka A , Mallery DL , et al. SARS-CoV-2 infects the brain choroid plexus and disrupts the blood-CSF barrier in human brain organoids. Cell Stem Cell. 2020;27:951–961.e955. 33113348 10.1016/j.stem.2020.10.001 PMC7553118

[5] Arabi YM , Harthi A , Hussein J , et al. Severe neurologic syndrome associated with Middle East respiratory syndrome corona virus (MERS-CoV). Infection. 2015;43:495–501. 25600929 10.1007/s15010-015-0720-y PMC4521086

[6] Gu J , Gong E , Zhang B , et al. Multiple organ infection and the pathogenesis of SARS. J Exp Med. 2005;202:415–24. 16043521 10.1084/jem.20050828 PMC2213088

[7] Lau KK , Yu WC , Chu CM , Lau ST , Sheng B , Yuen KY. Possible central nervous system infection by SARS coronavirus. Emerg Infect Dis. 2004;10:342–4. 15030709 10.3201/eid1002.030638 PMC3322928

[8] Yeh EA , Collins A , Cohen ME , Duffner PK , Faden H. Detection of coronavirus in the central nervous system of a child with acute disseminated encephalomyelitis. Pediatrics. 2004;113:e73–76. 14702500 10.1542/peds.113.1.e73

[9] Arbour N , Day R , Newcombe J , Talbot PJ. Neuroinvasion by human respiratory coronaviruses. J Virol. 2000;74:8913–21. 10982334 10.1128/jvi.74.19.8913-8921.2000 PMC102086

[10] Amanat M , Rezaei N , Roozbeh M , et al. Neurological manifestations as the predictors of severity and mortality in hospitalized individuals with COVID-19: a multicenter prospective clinical study. BMC Neurol. 2021;21:116. 33726699 10.1186/s12883-021-02152-5 PMC7960879

[11] Mao L , Jin H , Wang M , et al. Neurologic manifestations of hospitalized patients with coronavirus disease 2019 in Wuhan, China. JAMA Neurol. 2020;77:683–90. 32275288 10.1001/jamaneurol.2020.1127 PMC7149362

[12] Chou SH , Beghi E , Helbok R , et al. Global incidence of neurological manifestations among patients hospitalized with COVID-19 - a report for the GCS-NeuroCOVID Consortium and the ENERGY Consortium. JAMA Netw Open. 2021;4:e2112131. 10.1001/jamanetworkopen.2021.12131 PMC8114143 33974053

[13] Maury A , Lyoubi A , Peiffer-Smadja N , de Broucker T , Meppiel E. Neurological manifestations associated with SARS-CoV-2 and other coronaviruses: a narrative review for clinicians. Rev Neurol. 2021;177:51–64. 33446327 10.1016/j.neurol.2020.10.001 PMC7832485

[14] Fragiel M , Miró Ò. , Llorens P , et al. Incidence, clinical, risk factors and outcomes of Guillain-Barré in Covid-19. Ann Neurol. 2021;89:598–603. 33295021 10.1002/ana.25987

[15] Ellul MA , Benjamin L , Singh B , et al. Neurological associations of COVID-19. Lancet Neurol. 2020;19:767–83. 32622375 10.1016/S1474-4422(20)30221-0 PMC7332267

[16] Correia AO , Feitosa PWG , Moreira JLS , et al. Neurological manifestations of COVID-19 and other coronaviruses: a systematic review. Neurol Psychiatr Brain Res. 2020;37:27–32. 10.1016/j.npbr.2020.05.008 PMC7261450 32834527

[17] Leeb RT , Price S , Sliwa S , et al. COVID-19 trends among school-aged children - United States, March 1-September 19, 2020. MMWR Morb Mortal Wkly Rep. 2020;69:1410–5. 33001869 10.15585/mmwr.mm6939e2 PMC7537558

[18] PHAC. Coronavirus disease 2019 (COVID-19): epidemiology update [online], Available at: https://health-infobase.canada.ca/covid-19/epidemiological-summary-covid-19-cases.html, Accessed March 14, 2021.

[19] Ray STJ , Abdel-Mannan O , Sa M , et al. Neurological manifestations of SARS-CoV-2 infection in hospitalised children and adolescents in the UK: a prospective national cohort study. Lancet Child Adolesc Health. 2021 10.1016/S2352-4642(21)00193-0 PMC8279959 34273304

[20] LaRovere KL , Riggs BJ , Poussaint TY , et al. Neurologic involvement in children and adolescents hospitalized in the United States for COVID-19 or multisystem inflammatory syndrome. JAMA Neurol. 2021;78:536–47. 10.1001/jamaneurol.2021.0504 PMC7936352 33666649

[21] Singer TG , Evankovich KD , Fisher K , Demmler-Harrison GJ , Risen SR. Coronavirus infections in the nervous system of children: a scoping review making the case for long-term neurodevelopmental surveillance. Pediatr Neurol. 2021;117:47–63. 33676141 10.1016/j.pediatrneurol.2021.01.007 PMC7988307

[22] Garazzino S , Montagnani C , Donà D , et al. Multicentre Italian study of SARS-CoV-2 infection in children and adolescents, preliminary data as at 10 April 2020. Euro Surveill. 2020;25. 10.2807/1560-7917.ES.2020.25.18.2000600 PMC7219028 32400362

[23] Harris PA , Taylor R , Minor BL , et al. The REDCap consortium: building an international community of software platform partners. J Biomed Inform. 2019;95:103208. 31078660 10.1016/j.jbi.2019.103208 PMC7254481

[24] WHO. Multisystem inflammatory syndrome in children and adolescents with COVID-19. WHO; 2020.

[25] Fink EL , Robertson CL , Wainwright MS , et al. Prevalence and risk factors of neurologic manifestations in hospitalized children diagnosed with acute SARS-CoV-2 or MIS-C. Pediatr Neurol. 2021;128:33–44. 35066369 10.1016/j.pediatrneurol.2021.12.010 PMC8713420

[26] Antoon JW , Grijalva CG , Thurm C , et al. Factors associated with COVID-19 disease severity in US children and adolescents. J Hosp Med. 2021;16:603–10. 34613896 10.12788/jhm.3689 PMC8494279

[27] Kurd M , Hashavya S , Benenson S , Gilboa T. Seizures as the main presenting manifestation of acute SARS-CoV-2 infection in children. Seizure. 2021;92:89–93. 34481322 10.1016/j.seizure.2021.08.017 PMC8397499

[28] Panda PK , Sharawat IK , Panda P , Natarajan V , Bhakat R , Dawman L. Neurological complications of SARS-CoV-2 infection in children: a systematic review and meta-analysis. J Trop Pediatrics. 2021;67:fmaa070. 10.1093/tropej/fmaa070 PMC7499728 32910826

[29] Lu L , Xiong W , Liu D , et al. New onset acute symptomatic seizure and risk factors in coronavirus disease 2019: a retrospective multicenter study. Epilepsia. 2020;61:e49–e53. 32304092 10.1111/epi.16524 PMC7264627

[30] Romero-Sánchez CM , Díaz-Maroto I , Fernández-Díaz E , et al. Neurologic manifestations in hospitalized patients with COVID-19: the ALBACOVID registry. Neurology. 2020;95:e1060–e1070. 32482845 10.1212/WNL.0000000000009937 PMC7668545

[31] Zheng J , Chen F , Wu K , et al. Clinical and virological impact of single and dual infections with influenza A (H1N1) and SARS-CoV-2 in adult inpatients. PLOS Neglect Trop Dis. 2021;15:e0009997. 10.1371/journal.pntd.0009997 PMC8659415 34843492

[32] Song E , Zhang C , Israelow B , et al. Neuroinvasion of SARS-CoV-2 in human and mouse brain. J Exp Med. 2021;218:e20202135. 10.1084/jem.20202135 PMC7808299 33433624

[33] Yan R , Zhang Y , Li Y , Xia L , Guo Y , Zhou Q. Structural basis for the recognition of SARS-CoV-2 by full-length human ACE2. Science (New York, NY). 2020;367:1444–8. 10.1126/science.abb2762 PMC7164635 32132184

[34] Xiang P , Xu X , Lu X , et al. Case report: identification of SARS-CoV-2 in cerebrospinal fluid by ultrahigh-depth sequencing in a patient with coronavirus disease 2019 and neurological dysfunction. Front Med. 2021;8:629828. 10.3389/fmed.2021.629828 PMC7937706 33693018

[35] Domingues RB , Mendes-Correa MC , de Moura Leite FBV , et al. First case of SARS-COV-2 sequencing in cerebrospinal fluid of a patient with suspected demyelinating disease. J Neurol. 2020;267:3154–6. 32564153 10.1007/s00415-020-09996-w PMC7305694

[36] Chu H , Chan JF , Yuen TT , et al. Comparative tropism, replication kinetics, and cell damage profiling of SARS-CoV-2 and SARS-CoV with implications for clinical manifestations, transmissibility, and laboratory studies of COVID-19: an observational study. Lancet Microbe. 2020;1:e14–e23. 32835326 10.1016/S2666-5247(20)30004-5 PMC7173822

[37] Beslow LA , Linds AB , Fox CK , et al. Pediatric ischemic stroke: an infrequent complication of SARS-CoV-2. Ann Neurol. 2021;89:657–65. 33332607 10.1002/ana.25991

[38] Majmundar N , Ducruet A , Prakash T , Nanda A , Khandelwal P. Incidence, pathophysiology, and impact of coronavirus disease 2019 (COVID-19) on acute ischemic stroke. World Neurosurg. 2020;142:523–5. 32987584 10.1016/j.wneu.2020.07.158 PMC7510414

[39] Varatharaj A , Thomas N , Ellul MA , et al. Neurological and neuropsychiatric complications of COVID-19 in 153 patients: a UK-wide surveillance study. Lancet Psychiatr. 2020;7:875–82. 10.1016/S2215-0366(20)30287-X PMC7316461 32593341

