# Supplementary material for: Neurological involvement in hospitalized children with SARS-CoV-2 infection: a multinational study
Source: Can J Neurol Sci. 2023 Jan 4:1–10. doi: 10.1017/cjn.2022.347 (PMC9947047; doi:10.1017/cjn.2022.347)
Supplement: Supplementary file 1 [file cjnsup.zip › S031716712200347Xsup001.docx]

**Supplementary Table 3.** Clinical features of children with demyelinating syndromes (n=1) and children with neurological syndromes/signs other than acute encephalopathy or seizures (n=4; please refer to Figure 1).

| **Age (year) at presentation/ Sex** | **Underlying condition** | **Primary reason for admission** | **Neurological manifestation** | **MRI findings** | **CSF and MOG/AQP4 results** |
| --- | --- | --- | --- | --- | --- |
| 16/F | Asthma,   IgA nephropathy | Abnormal movements | - High amplitude, non-rhythmic, jerk-like left arm, head, leg jerking  - Dysarthria | Brain MRI: Incidental finding of pituitary lesion concerning for cystic microadenoma vs Rathke's cyst with hemorrhagic component. | CSF: Not done  MOG/AQP4 ab: not done |
| 2.9/M | None | Bilateral lower limb weakness | - Progressive lower limb weakness  symmetric weakness in the context of hyporeflexia - Confusion - Agitation | Brain MRI: T2/FLAIR signal hyperintensity in the deep cerebellar hemispheres, more pronounced on the right. Possible subtle enhancement of the lower cranial nerves.   Spine MRI: Marked abnormal enhancement of the cauda equina associated with mild thickening of the nerve roots. | CSF:  - WBC 4 - RBC 282 - Protein 2.22 g/L - Glucose 3.7 mmol/L - % neutrophils ND - % lymphocytes ND - OCB negative  MOG/AQP4 ab: negative |
| 13/F | None | Weakness in right foot | Weakness in right foot (MRC 2) | Not done | CSF: Not done  MPG/AQP4 ab: Not done |
| 2/F | None | Ataxia | Ataxia | Not done | CSF: Not done  MOG/AQP4 ab: Not done |
| 10/F | None | Gait abnormalities, paraesthesias | Paresthesias below neck, normal sensory examination. Brisk reflexes | Brain MRI: Limited sequences, unremarkable.    Spine MRI: Subtle signal hyperintensity within the upper cervical spinal cord | CSF:  - WBC 3 - RBC 3 - Protein 0.15g/L - Glucose 3.1 - % neutrophils 1 - % monocytes 38 - % lymphocytes 61 - OCB negative  MOG/AQP4: Not done |
