# Supplementary material for: Neurological involvement in hospitalized children with SARS-CoV-2 infection: a multinational study
Source: Can J Neurol Sci. 2023 Jan 4:1–10. doi: 10.1017/cjn.2022.347 (PMC9947047; doi:10.1017/cjn.2022.347)
Supplement: Supplementary file 1 [file cjnsup.zip › S031716712200347Xsup002.docx]

**Supplementary Table 1.** Demographics and clinical characteristics of study cohort stratified by country

|  | **All patients**  **(n=697)** | **Canada (n=435)** | **Costa Rica (n=205)** | **Iran (n=57)** |
| --- | --- | --- | --- | --- |
| **Demographics** |  |  |  |  |
| Age at admission, years | 4 (0.9-10.5) | 6 (1.3-13.8) | 2.7 (0.5-7.5) | 2.1 (0.7-5.3) |
| Male | 403 (58%) | 244(56%) | 126 (61%) | 33 (58%) |
| Female | 294 (42%) | 191 (44%) | 79 (38%) | 24 (42%) |
| **Underlying conditions** |  |  |  |  |
| Previously healthy | 390 (56%) | 234 (54%) | 126 (61%) | 30 (52%) |
| Preterm birth (< 37 weeks GA) | 45 (6%) | 39 (9%) | 6 (3%) | 0 |
| Congenital immunodeficiency | 4 (0.6%) | 3 (0.7%) | 0 | 1 (2%) |
| Cancer | 36 (5%) | 13 (3%) | 16 (8%) | 7 (12%) |
| Cardiovascular disease | 19 (3%) | 12 (3%) | 4 (2%) | 3 (5%) |
| Asthma | 46 (7%) | 22 (5%) | 22 (11%) | 2 (3%) |
| Cystic fibrosis | 2 (0.3%) | 2 (0.5%) | 0 | 0 |
| Chromosomal disorder | 11 (2%) | 8 (2%) | 3 (1%) | 0 |
| Diabetes | 11 (2%) | 9 (2%) | 1 (0.5%) | 1 (2%) |
| Hypertension | 9 (1%) | 5 (1%) | 4 (2%) | 0 |
| Obesity | 57 (8%) | 44 (10%) | 12 (6%) | 1 (2%) |
| Neurological disorder | 74 (10%) | 54 (12%) | 15 (7%) | 5 (9%) |
| Seizure disorder | 40 (6%) | 30 (7%) | 8 (4%) | 2 (3%) |
| Developmental delay/autism | 38 (5%) | 31 (7%) | 7 (3%) | 0 |
| Cerebral palsy | 8 (1%) | 4 (1%) | 1 (0.5%) | 3 (5%) |
| ≥ 2 comorbid conditions | 92 (13%) | 67 (15%) | 22 (11%) | 3 (5%) |
| Met MIS-C criteria | 140 (20%) | 102 (23%) | 30 (15%) | 8 (14%) |
| ICU | 176 (25%) | 126 (29%) | 30 (15%) | 20 (35%) |
| Mechanical ventilation* | 114 (16%) | 72 (16%) | 25 (12%) | 17 (30%) |
| Duration of hospital stay, days | 4 (2-8) | 4 (2-8) | 4 (3-7) | 7 (6-10) |
| Any neurological manifestations | 147 (21%) | 91 (21%) | 44 (21%) | 12 (21%) |
| Headache only | 85 (12%) | 57 (13%) | 21 (10%) | 7 (12%) |

*Data available from 665 patients. Abbreviations: ICU, intensive care unit; MIS-C, multisystem inflammatory syndrome in children
