# Supplementary material for: Neurological involvement in hospitalized children with SARS-CoV-2 infection: a multinational study
Source: Can J Neurol Sci. 2023 Jan 4:1–10. doi: 10.1017/cjn.2022.347 (PMC9947047; doi:10.1017/cjn.2022.347)
Supplement: Supplementary file 1 [file cjnsup.zip › S031716712200347Xsup003.docx]

**Supplementary Table 2.** Comparison of non-headache neurological cases (n=62) and headache-only cases (n=85)

^1^Oseltamivir, Kaletra, Remdesivir
