# Supplementary material for: Neurological involvement in hospitalized children with SARS-CoV-2 infection: a multinational study
Source: Can J Neurol Sci. 2023 Jan 4:1–10. doi: 10.1017/cjn.2022.347 (PMC9947047; doi:10.1017/cjn.2022.347)
Supplement: Supplementary file 1 [file cjnsup.zip › S031716712200347Xsup004.docx]

Supplementary Figure. Cases of neurological manifestations that had potential pathogens detected in addition to SARS-CoV-2

^✭^Cases were not included in the analyses of cases deemed attributable to SARS-CoV-2

^✽^ One patient tested positive for RV/EV and parainfluenza 4

^▲^E. coli was detected in the CSF (n=2), urine (n=3). One patient had peritonitis due to E. coli and E. gallinarum

Abbreviations: EV/RV = entero/rhinovirus; RV = rhinovirus; WNV = West Nile virus; MRSA = methicillin-resistant Staphylococcus aureus; CMV = cytomegalovirus; NP = nasopharyngeal swab; CSF = cerebrospinal fluid; UTI = urinary tract infection
